# Martin Baumann, Christoph Gordalla: Gruppenarbeit: Methoden – Techniken – Anwendungen

**DOI:** 10.3205/zma000956

**Published:** 2015-05-13

**Authors:** Reinhard Putz

**Affiliations:** 1 LMU München, Anatomische Anstalt, München, Deutschland

## Recension

At first sight this is a book with an uncontroversial title. Who is not at times and under the most various of circumstances going to, or be obliged to, opt for group work? But its focus is on a topic the potential of which is often underestimated. It targets a wide range of readers, primarily in the field of academic instruction, but also in business and manufacturing. The central aspects of group work are presented in nine systematic chapters, which ensures a surprisingly easy navigation of a plethora of methods and techniques.

The impartial reader feels appreciated already in the preface, which provides a suggestion for structured approach to the abundance of material provided. The first four chapters are especially helpful for the beginner, as they provide guidelines on fundamental questions of group work, from determining goals to practical application to a discussion of the role of the group leader or moderator. There is a brief introductory analysis of the processes of group dynamics, which might help future practitioners of these approaches avoid awkward errors.

The authors attribute enough importance to group dynamics to devote a separate chapter to the problems of group formation and collaboration within the group. After all, it is in the initial phase that the foundations are laid for future successful cooperation.

The two central – the main portion of the book – deal with techniques for searching and finding ideas and developing concepts. Each subchapter opens with a clear statement of goals, which is followed by a meticulous description of the relevant technique and a comparison of applicable pros and cons. Initial orientation is made easy by brief and usually poignant surveys. The chapters provide an exhaustive and lucid presentation of a broad spectrum of – often widely diverging – techniques. This practical overview is an invitation to try out new processes and compare their efficiency.

The subject of the book is transcended in those chapters that deal with learning techniques on the one hand and presentation methods on the other. They provide clear and encouraging suggestions for shared learning as a particular application of group work. The book concludes with a catalog of suggestions for lecture planning and PowerPoint presentations. These are somewhat cursory, but they provide a goal-oriented complement of the topic. Together with the opening chapters, the two concluding chapters provide the framework for a list of examples of group work. By that, the authors are able to demonstrate that in order to successfully employ various group work techniques both in educational and organizational settings, one has to be able to rely on a broad foundational understanding of the underlying processes.

This book is useful in more than one way. For the experienced leader, it will provide a number of interesting new ideas, but also proposals for rethinking routinely used techniques and examining their efficiency regarding various purposes. For the beginner, it offers a plethora of suggestions, as well as sound guidance in finding the most apt technique for a given situation. Finally, it warrants mention that the book is consistent to the smallest detail and extremely well-structured. 

## Competing interests

The author declares that he has no competing interests.

